# High-Tribological-Performance Polymer Nanocomposites: An Approach Based on the Superlubricity State of the Graphene Oxide Agglomerates

**DOI:** 10.3390/polym13142237

**Published:** 2021-07-08

**Authors:** Eder H. C. Ferreira, Angela Aparecida Vieira, Lúcia Vieira, Guilhermino J. M. Fechine

**Affiliations:** 1 Mackenzie Institute for Research in Graphene and Nanotechnologies-MackGraphe, Mackenzie Presbyterian University, Rua da Consolação, 896, São Paulo 01302-907, Brazil; eder-henrique2011@hotmail.com; 2 Institute of Research and Development-IP&D, University of Paraiba Valley-Univap, Av, Shishima Hifumi, 2911-Urbanova, São José dos Campos 12244-000, Brazil; angela.1002@hotmail.com (A.A.V.); lucia.vieira@univap.br (L.V.)

**Keywords:** UHMWPE, tribological performance, graphene oxide, superlubricity phenomenon, nanocomposite

## Abstract

Here, nanocomposites of high-molecular-weight polyethylene (HMWPE) and HMWPE-UHMWPE (80/20 wt.%) containing a low amount of multilayer graphene oxide (mGO) (≤0.1 wt.%) were produced via twin-screw extrusion to produce materials with a higher tribological performance than UHMWPE. Due to the high viscosity of both polymers, the nanocomposites presented a significant concentration of agglomerates. However, the mechanical (tensile) and tribological (volumetric loss) performances of the nanocomposites were superior to those of UHMWPE. The morphology of the nanocomposites was investigated using differential scanning calorimetry (DSC), microtomography, and transmission electron microscopy (TEM). The explanation for these results is based on the superlubricity phenomenon of mGO agglomerates. It was also shown that the well-exfoliated mGO also contained in the nanocomposite was of fundamental importance as a mechanical reinforcement for the polymer. Even with a high concentration of agglomerates, the nanocomposites displayed tribological properties superior to UHMWPE’s (wear resistance up to 27% higher and friction coefficient up to 57% lower). Therefore, this manuscript brings a new exception to the rule, showing that agglomerates can act in a beneficial way to the mechanical properties of polymers, as long as the superlubricity phenomenon is present in the agglomerates contained in the polymer.

## 1. Introduction

Ultra-high-molecular-weight polyethylene (UHMWPE) is an engineering polymer that has excellent tribological properties. Its semicrystalline structure and high tie chain density give this polymer a very high abrasive wear resistance and low friction coefficient compared to other polymers [1,2]. UHMWPE is widely found in applications where good tribological performance is required, such as biomedical implants, bearings and gears, coating the surface of dump truck buckets, production belts and feed hoppers [3]. UHMWPE is in contact with materials with high wear resistance and hardness (steel, titanium, glass, sand, ores, etc.) in these applications, which can cause abrasive wear on the UHMWPE, reducing its useful life. However, the development of new materials with better wear resistance than UHMWPE is quite attractive for several sectors of the industry.

The addition of nanoparticles, carbon nanotubes (CNTs), graphene oxide (GO), graphene (Gr), nanoclays, etc. to UHMWPE (nanocomposites) has been sought as alternatives to develop new materials with better mechanical performance [4,5,6,7]. Although some authors have reported good performances of the UHMWPE nanocomposites produced, other authors have shown that UHMWPE as a matrix may cause the formation of a high agglomerates content of CNT and Gr, damaging the mechanical properties of the nanocomposite [7]. This is because UHMWPE is not processable using conventional mixing techniques, due to its high viscosity, only produced using the powder sintering technique [3]. This technique has a poor mixing capacity, resulting in a high content of agglomerates placed at the boundary of the sintered powder, which will prevent the molecular diffusion between the particles during sintering, and it will act as a stress concentrator during the mechanical test [3,7]. The development of nanocomposites, as an alternative, has been using low-molecular-weight polyethylene matrices (high-density polyethylene (HDPE), low-density polyethylene (LDPE), linear low-density polyethylene (LLDPE)) [8,9,10]. Despite achieving a good mixture of the nanofiller in these matrices due to using conventional melt mixing processing techniques, the material obtained still did not present a very high tribological performance compared to UHMWPE [11].

Ferreira et al. (2019 and 2020) recently studied the mechanical tensile properties of the high-molecular-weight polyethylene (HMWPE) nanocomposite containing 0.1 wt.% of mGO and graphite oxide (GrO), and they observed that due to the high viscosity of the matrix, this nanocomposite and composite had a high content of agglomerates but with satisfactory ultimate tensile properties [12]. Their research showed that the agglomerates are included in the HMWPE matrix and are not segregated at the boundary of the powders, not weakening the material. Another essential feature of this system is that during the processing of the HMWPE nanocomposite, the polymer flow inside the extruder guides the agglomerates in the same direction, conducting the mGO agglomerated flakes, so they are organised parallel to each other, similar to playing cards. They also reported that agglomerates arranged in this way can act as lubricants by slipping between the agglomerated flakes (superlubricity phenomenon) [12,13]. Slips from the agglomerates can help the polymer achieve greater tensile toughness in the solid state and reduce viscosity in the molten state as they are present in small amounts [12,13]. This study was a paradigm shift in the science of polymeric composites because it showed that agglomerates could act beneficially to the properties of polymers and not just as a defect. Other authors have also reported the influence of the lubricating behaviour of mGO, graphite, HBN, and MSO2 agglomerates on the properties of polymers [13,14,15,16,17,18,19,20,21].

Few-layer and multilayer graphene oxide (fGO and mGO, respectively) are nanoparticles with desirable properties to use as fillers for polymeric nanocomposites. Cao et al. (2015) reported that the elastic modulus and tensile strength of mGO can vary between 103 and 291 GPa, and 4 and 12 GPa, respectively, with the thickness ranging from 24 to 75 nm. These values are higher than those of conventional fillers used for the production of polymeric composites [22].

A new strategy was followed here to obtain new materials with better tribological performance than that of UHMWPE. HMWPE is a polyethylene matrix with intermediate molecular weight, i.e., it presents better tribological properties than low-molecular-weight polyethylene and a better processability than UHMWPE. In a previous study [12,13], despite the processing difficulty (mixing) of HMWPE nanocomposites in relation to HDPE, LDPE and LLDPE nanocomposites, it was observed that the agglomerates contained in HMWPE might present a superlubricity phenomenon, being beneficial to the properties of the polymer. A new study based on the superlubricity phenomenon of nanofiller agglomerates and its consequences on the tribological properties of polyethylene matrix nanocomposites was carried out here. For this study, several new nanocomposites, HMWPE and HMWPE-UHMWPE (80/20 wt.%) nanocomposites containing 0.01, 0.05 and 0.1% of multilayer graphene oxide (mGO), were produced using melt mixing via twin-screw extrusion. In addition, the importance of the agglomerates for the development of new high-performance materials for tribological applications was shown. The mechanical tensile properties and abrasive wear resistance of nanocomposites were evaluated and discussed based on the Ratner–Lancaster correlation. In addition, the tribological properties of UHMWPE were studied and compared with the new materials produced.

## 2. Materials and Methods

### 2.1. Characterisation of Fillers

The mGO used here was the same used by Ferreira et al. (2019) [13]. The characterisations of the particle have been meticulously detailed in their work.

### 2.2. Processing of Nanocomposites

Two polymers were used: High-molecular-weight polyethylene (HMWPE) with a number-average molecular weight ($\bar{M}$n) of 1.48 × 10^5^ g/mol and ultra-high-molecular-weight polyethylene (UHMWPE) with $\bar{M}$n of 3.0 × 10^6^ g/mol. Braskem supplied both polymers.

The studied materials were HMWPE, HMWPE-mGO (0.01, 0.05 and 0.1 wt.%), HMWPE-UHWMPE (80/20 wt.%) blend, HMWPE-UHMWPE (80/20 wt.%)-mGO (0.01, 0.05 and 0.1 wt.%) and UHMWPE. HMWPE and HMWPE-mGO (0.1 wt.%) were the same materials studied by Ferreira et al. (2019) [13] and Ferreira et al. (2020) [12]. The HMWPE-UHMWPE (20 wt.%) blend and UHMWPE were the same studied by Ferreira and Fechine. (2020 and 2021) [11,23]. With the exception of the UHMWPE, all materials were processed in a twin-screw extruder. The materials were processed in a twin-screw extruder L/D = 40 (Process 11, ThermoScientific) at a screw velocity of 150 rpm, operating with a temperature profile in zone 1 at 115 °C, zone 2 at 170 °C, zones 3–7 at 200 °C, and with a die at 200 °C with a feed rate of 3 g/min. The HMWPE, HMWPE-UHMWPE blend, and their nanocomposites, due to their high viscosity, were processed without the extrusion die, to allow them to be processed.

The moulding conditions used here were based on previous studies [13,23]. The best moulding conditions for all materials were in a Hot Hydraulic Press (SL-11/20, Solab, São Paulo, Brazil), operating at a temperature of 200 °C on the top and bottom plate and subjected to 6 tonnes of pressure for 60 min to achieve maximum mechanical performance [11,23]. Under these conditions, the maximum mixing degree between polymers can be achieved [23]. These moulding conditions also provide good compatibility between the polymer and nanofiller, even with the opposite chemical nature of polyethylene and mGO (apolar and polar, respectively); this happened due to the thermal reduction of mGO [13]. 

### 2.3. Characterisation of Nanocomposites

X-ray microtomography: Pieces at least 2 mm × 2 mm × 1.2 mm from specimens were used for this characterisation. Samples were analysed in a 3D X-ray Microscopy (Skyscanner 1272, Bruker, Billerica, MA, USA), using 20 kV and a 175 µA X-ray source, with a final image resolution of 2 µm/pixel.

Transmission electron microscopy (TEM): TEM imaging was performed on a Tecnai G2-20 Fei SuperTwin microscope (FEI Company, Hillsboro, OR, USA) at 200 kV. Ultramicrotome samples 60 nm in thickness were collected on top of 200 mesh copper grids.

Tensile test: The stress–strain tests of HMWPE and HMWPE-UHMWPE (80/20 wt.%) and its nanocomposites were performed at a deformation rate of 20 mm/min, at room temperature on a Zwick/Roell Z100 testing machine (Zwick/Roell, Ulm, Germany). Tests were performed on 7 specimens for each type of sample. The sample specification followed the ASTM D 638-14 Type V standard.

Differential scanning calorimetry (DSC): The analyses of HMWPE and HMWPE-UHMWPE (80/20 wt.%), their nanocomposites, and UHMWPE were carried out on a DSC-60 PLUS—Shimadzu (Shimadzu, Barueri, São Paulo, Brazil). The parameters used were a 10 °C/min heating rate from 30 °C to 200 °C under a nitrogen atmosphere. The sample weight was about 7.5 mg, and a lid crucible of aluminium was used.

Rheological test in oscillatory flow (Anton Paar 102 rheometer, Anton Paar, São Paulo, Brazil): The test was conducted using plate/plate geometry, at 200 °C, angular velocities of 0.01 to 100 rad/s, and a deformation of 1% (within the linear viscoelasticity regime) under an inert atmosphere. The samples were left in thermal equilibrium for 15 min immediately before the test, and testing lasted for 2 h.

Abrasive test: The abrasive wear resistance of the HMWPE, UHMWPE, HMWPE-UHMWPE (80/20 wt.%), HMWPE-mGO (0.01, 0.05 and 0.1 wt.%) and HMWPE-UHMWPE (80/20 wt.%)-mGO (0.01, 0.05 and 0.1 wt.%) were evaluated using a pin-on-drum abrasive wear test according to ISO 4649:2017. The parameters used were a 10 N normal force, 40 m sliding distance, 0.15 m drum diameter and 40 rpm drum rotation speed. Medium-sized alumina sandpaper of 265 µm was used. Tests were performed on 5 specimens for each type of sample. The results were expressed in volumetric loss (mm^3^) using the equation below [24]:

(1) $$\Delta V=\frac{\Delta m*\Delta mpconst}{\rho *\Delta mp}\;\;\;$$

where Δ*m* is the difference between the polymer mass before and after the abrasive test, Δ*mpconst* (200 mg) is a constant based on the wear of the standard material, ρ is the density of the polymer, and Δmp is the mass loss of the standard material after the abrasive test. The density (ρ) of the materials was calculated based on the crystallinity degree of samples obtained from DSC curves. The crystallinity degree obtained from the DSC curves is in the Supporting Information. The equation used to calculate the density was [25]:

(2) $$\rho =\frac{-\rho_{c}\rho_{a}}{Xc*\left(\rho_{c}-\rho_{a}\right)-\rho_{c}}\;$$

where Xc is the crystallinity degree calculated from DSC curves, $\rho_{c}$ is 1.00 g/cm^3^ referring to the density of crystalline solid, and $\rho_{a}$ is 0.85 g/cm^3^ referring to the density of the amorphous solid. 

Scanning electron microscopy (SEM): The worn surface of specimens after the abrasive test of the HMWPE, HMWPE-mGO (0.1 wt.%), HMWPE-UHMWPE (80/20 wt.%) and HMWPE-UHMWPE (80/20 wt.%)-mGO (0.1 wt.%) was analysed on a HITACHI TM3000 tabletop microscope (Hitachi, Chiyoda, Tokyo, Japan) at 15 keV.

Friction coefficient analysis: The HMWPE, UHMWPE, HMWPE-UHMWPE (80/20 wt.%), HMWPE-mGO (0.01, 0.05 and 0.1 wt.%) and HMWPE-UHMWPE (80/20 wt.%)-mGO (0.01, 0.05 and 0.1 wt.%) were evaluated using a Bruker Ultra Micro Tribometer (Bruker, Billerica, MA, USA). The parameters were: An SS316L counterface ball 4 mm in diameter was used as a counter-body with a fresh surface on each test. The oscillation frequency of the counterface ball was controlled at 1 Hz under a normal force of approximately 15 N, a velocity of 20 mm/s, and a horizontal displacement of 10 mm for 300 s. Tests were performed on 3 specimens for each type of sample.

## 3. Results and Discussions

It is known that agglomerates act as a defect in the polymer, preventing the total wettability of the filler by the polymer and being the leading cause of premature fracture during mechanical tests [26,27]. Images of microtomography and TEM are presented to demonstrate that two types of structures (nonagglomerated and agglomerated) can be generated in the nanocomposites produced here.

**Figure 1:**
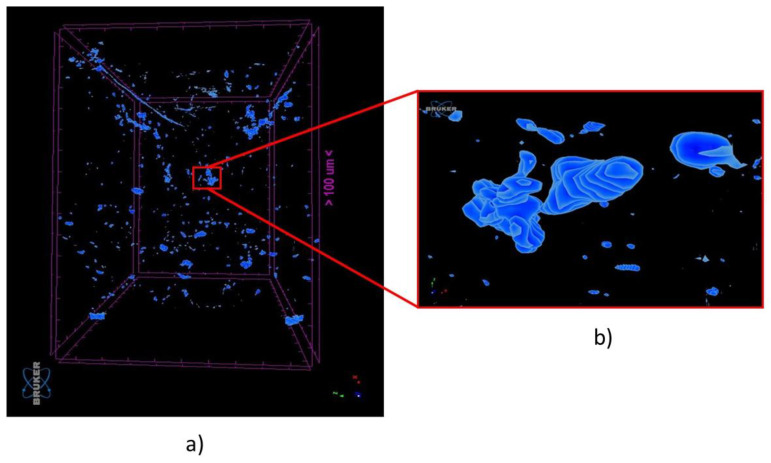
Image of mGO agglomerates (**a**,**b**) contained in HMWPE-UHMWPE. The images were obtained from analyses of HMWPE-UHMWPE (20 wt.%)-mGO (0.1 wt.%) nanocomposites using X-ray microtomography.

Figure 1a,b show the microtomography images of the HMWPE-UHMWPE (80/20 wt.%)-mGO (0.1 wt.%) nanocomposite. Note that this material possesses a significant content of agglomerates (blue region). This is due to the high viscosity of HMWPE (continuous phase), which makes it difficult to disperse and distribute mGO particles during the processing of the nanocomposites. The mixture becomes even poorer with the addition of 20% UHMWPE, which further increases the viscosity of the system. Looking only at Figure 1a, the predictions for the mechanical properties of these nanocomposites are quite unsatisfactory due to the high agglomerate content. However, as is seen later, the opposite has occurred. Despite the high agglomerate content, as shown in Figure 1b, the mGO flakes are agglomerated in an organised manner, similar to playing cards. This structure favours the slip between the flakes (superlubricity phenomenon), making the agglomerates act as lubricants in the solid and molten state of the polymer [12,13]. The superlubricity phenomenon of mGO appears due to the high amount of out-of-register flake–flake contacts present in the agglomerates [13]. The friction between these contacts is extremely low, giving rise to superlubricity [13,28,29,30]. The organised way that the agglomerates present themselves allows them to not act as a defect during the polymer test but instead as a mechanism of energy dissipation due to the superlubricity phenomenon, as is shown later [12].

**Figure 2:**
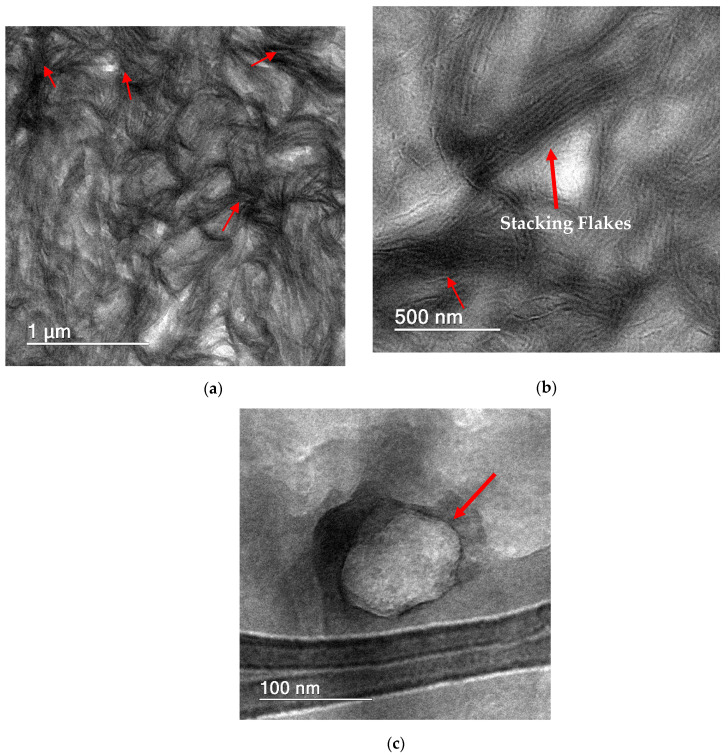
TEM images of agglomerated mGO (**a**,**b**) and nonagglomerated mGO (**c**) contained in the HMWPE.

Figure 2 shows TEM images of the agglomerated mGO (a,b) and nonagglomerated mGO (c) in the HMWPE-mGO nanocomposite (0.1 wt.%). As mentioned earlier, Figure 1 and Figure 2 indicate that the nanocomposites produced here present regions with agglomerated and nonagglomerated mGO particles. However, it is reasonable to think that two possible mechanisms can reinforce the polymer during mechanical testing. The agglomerated mGO acts through the slipping between the flakes (superlubricity phenomenon), and the nonagglomerated mGO acts as a mechanical reinforcement due to the adhesion of the polymer-filler interphase [13]. 

The mechanical tensile properties of nanocomposites and how these two mechanisms can act differently in the stress–strain curve are presented. Table 1 shows the values of the mechanical properties of the nanocomposites obtained from the tensile test. It is observed that the elastic modulus of the nanocomposites was lower or equal compared to neat HMWPE. It is commonly expected that the elastic modulus of the polymer increases with the addition of fillers, as they present a higher stiffness than the matrix. In nanoparticles, such as mGO, the increase in the elastic modulus of the polymer should be even more significant due to its high surface area that promotes a higher number of polymer-filler interphases. The mGO, when very well exfoliated inside the polymer matrix, significantly increases the elastic modulus of the nanocomposite, but it was not observed here [20]. The low elastic modulus of the nanocomposites can be attributed to the high content of agglomerates present and the superlubricity phenomenon. Slipping between the flakes contributes to energy dissipation and reduces the elastic modulus [12,13]. The amount of well-exfoliated mGO present in the matrix that can increase the elastic modulus through the adhesion of the polymer-filler interphase is insufficient to make the nanocomposite stiffer (greater elastic modulus) [12,13]. Other authors have reported a reduction in the elastic modulus in filled nanocomposites (mGO, graphene oxide, and graphite) that present the superlubricity phenomenon [12,16,31].

In the case of the HMWPE-UHMWPE (80/20 wt.%), there is one more factor contributing to the low values of the elastic modulus. The UHMWPE phase presents a low degree of crystallinity and, consequently, a low elastic modulus, resulting in low-modulus nanocomposites [23,32]. Comparing the HMWPE-UHMWPE (80/20 wt.%) blend and its nanocomposites, an increase in the elastic modulus is observed with the addition of mGO. This is due to the nucleating action of the mGO, which increases the crystallinity degree of the blend, increasing the elastic modulus. Table S1 shows the values of Xc obtained from the DSC analysis (Figure S1 shows DSC curves) of all materials studied here. The HMWPE-UHMWPE (80/20 wt.%)-mGO nanocomposites showed a higher crystallinity degree than the HMWPE-UHMWPE (80/20 wt.%) blend, thus justifying its largest modulus.

Table 1 also shows the values of stress at breaking-$\tau_{b}$ (a) and strain at breaking-$\varepsilon_{b}$ (b) of the nanocomposites obtained from tensile test curves. It is observed that the stress and strain at breaking of the HMWPE-mGO and HMWPE-UHMWPE (80/20 wt.%)-mGO nanocomposites were higher than those of HMWPE. Young et al. (2012) reported that the addition of graphene or graphene oxide to the polymers commonly increases its E and $\tau_{b}$, to the detriment of reducing its $\varepsilon_{b}$ [33]; however, it was not observed here. Ferreira et al. (2020) reported that the increase in the ultimate mechanical properties of HMWPE-mGO (0.1 wt.%) is due to the combined action of the well-exfoliated mGO and agglomerated mGO contained in the matrix [13]. The first acts by increasing the stress at breaking ($\tau_{b}$) through mechanical reinforcement, while the second acts by increasing the strain at breaking ($\varepsilon_{b}$) through the superlubricity phenomenon [12]. The HMWPE-UHMWPE (80/20 wt.%)-mGO nanocomposites showed a higher deformation than the HMWPE-mGO nanocomposites, except for HMWPE-mGO (0.01 wt.%), which had an atypical result. The stress at breaking values of the nanocomposites were similar. The higher deformation of the HMWPE-UHMWPE (80/20 wt.%)-mGO nanocomposites are due to the joint reinforcement action of the mGO and UHMWPE added. The UHMWPE phases act as an excellent reinforcement for the HMWPE matrix due to its very high tie chain density [23]. Comparing the HMWPE-UHMWPE (80/20 wt.%) blend with their respective nanocomposites, it is possible to observe that the addition of mGO increased the $\varepsilon_{b}$ of the blends, mainly to 0.1 wt.% of mGO, while the $\tau_{b}$ did not change.

**Figure 3:**
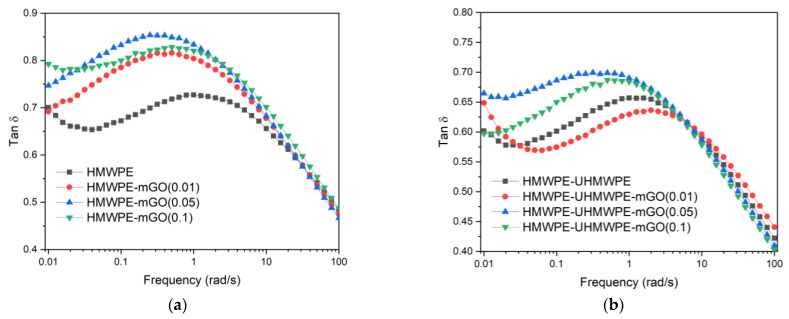
Loss factor tan δ of the HMWPE and its nanocomposites (**a**) and HMWPE-UHMWPE (80/20 wt.%) and its nanocomposites (**b**) obtained from the rheological test.

The contribution of the superlubricity phenomenon can be seen most clearly by evaluating the materials in the molten state [13]. Above the melting temperature (>150 ℃), the influence of crystallinity and the nucleating action of the particles is nonexistent [13]. Figure 3 shows the tan δ (loss modulus/storage modulus) of HMWPE and its nanocomposites (a), and the HMWPE-UHMWPE (80/20 wt.%) blend and its nanocomposites (b) in the molten state (200 °C) obtained from the rheological test. In general, nanocomposites present smaller tan δ than the neat polymer; that is, they present a higher solid-like behaviour due to the stiffening of the polymeric chain by the particle [34]. However, the opposite was observed in the nanocomposites presented here. At low frequencies (region below 1 rad/s where particle influence is more noticeable), it is observed that the tan δ of the nanocomposites were higher than the neat polymers. This indicates that the mGO agglomerate, through the superlubricity phenomenon, becomes HMWPE and HMWPE-UHMWPE (80/20 wt.%) with less solid-like behaviour [13]. The HMWPE-UHMWPE (80/20 wt.%)-mGO (0.05 wt.%) sample behaved very differently from the other nanocomposites; only at very low frequencies was the superlubricity phenomenon observed. Although less noticeable in the solid state, the superlubricity phenomenon of the agglomerates also affected the moduli of the polymers (semicrystalline and amorphous) [12].

**Figure 4:**
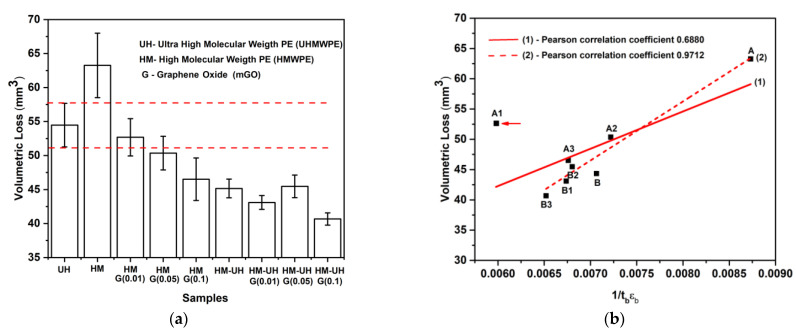
Results of volumetric loss after abrasive test (**a**) and Ratner–Lancaster correlation (**b**) of the UHMWPE [11], HMWPE (A) [11], HMWPE-mGO (0.01 (A1), 0.05 (A2) and 0.1 (A3) wt.%), HMWPE-UHMWPE (80/20 wt.%) (B) [11] and HMWPE-UHMWPE (80/20 wt.%)-mGO (0.01 (B1), 0.05 (B2) and 0.1 (B3) wt.%).

Figure 4a shows the volumetric loss after the abrasive test of the UHMWPE, HMWPE, HMWPE-mGO (0.01, 0.05 and 0.1 wt.%), HMWPE-UHMWPE (80/20 wt.%) and HMWPE-UHMWPE (80/20 wt.%)-mGO (0.01, 0.05 and 0.1 wt.%). The results prove that the addition of mGO to neat HMWPE and the HMWPE-UHMWPE (80/20 wt.%) blend significantly improves the abrasive wear resistance (volumetric loss reduced), mainly to 0.1 wt.% of mGO. The HMWPE-UHMWPE (80/20 wt.%)-mGO (0.1 wt.%) nanocomposite had the highest wear resistance among the analysed materials. This is due to the joint reinforcement action of the mGO and UHMWPE phases. Compared to UHMWPE, all nanocomposites showed less volumetric loss after the abrasive test, except for the HMWPE-mGO (0.01 wt.%) nanocomposite that had similar performance. It is important to emphasise again that the nanocomposites showed excellent performance, even with a high content of agglomerates. This reinforcement comes from the mGO agglomerates that can benefit the mechanical properties of polymers due to the superlubricity phenomenon, as long as a low filler content (0.1 wt.%) is added [12]. In addition, the HMWPE-UHMWPE (80/20 wt.%) blend showed a higher wear resistance than the UHMWPE when mGO was added. In this case, the high performance is correlated with the high mixing degree in the polyethylene–polyethylene interphase (healing phenomenon) achieved during moulding [11,23] and by the presence of mGO (as explained earlier).

The results of the tensile and abrasion test of the materials are now discussed correlating both tests. The elastic modulus (stiffness) often correlates with the wear resistance of the materials, especially for metallic and ceramic materials [35]. For polymeric composites, some authors reported that the increase in the elastic modulus of the polymer by the addition of fillers leads to increased wear resistance [36]. However, on the other hand, other authors have shown the opposite of that [36]. This manuscript shows that the nanocomposites showed a higher abrasive wear resistance than HMWPE, even with a smaller or similar elastic modulus compared to HMWPE (see Table 1). According to Lancaster (1968), a good strategy to improve the abrasive wear resistance of the polymer is to increases its tensile toughness. Lancaster suggested that the abrasive wear resistance of polymers is proportional to the product between the stress at breaking ($\tau_{b}$) and the strain at breaking ($\varepsilon_{b}$) [37]. This correlation between the tensile and abrasive test is called the “Ratner–Lancaster correlation”. Based on this correlation, several authors have predicted and obtained composites and nanocomposites with high wear resistance [38,39,40]. This paper also supports that this correlation is a good strategy to achieve higher wear resistance of the polymer by adding fillers, increasing the ultimate tensile properties and not stiffness. This can be seen by applying the Ratner–Lancaster correlation and linear regression to the data obtained from the abrasive (volumetric loss) and tensile (1/$\tau_{b}\varepsilon_{b}$) tests of the studied materials (Figure 4b). In Figure 4b, curve 1 refers to the linear regression considering all samples, while curve 2 refers to linear regression, which was applied without considering the HMWPE-mGO (0.01 wt.%) sample. As can be seen in Table 1 and Figure 4a, the HMWPE-mGO (0.01 wt.%) nanocomposite was the material that presented the highest values of stress and strain at breaking, but it was not the nanocomposite that presented the highest wear resistance. Figure 4b shows that this specific nanocomposite (point A1) is an atypical point concerning the others. The linear regression of all samples, represented by curve 1, shows that Pearson’s coefficient is 0.68, showing that there is a good correlation between the abrasive test and ultimate tensile properties, even considering the atypical point A1. Disregarding the HMWPE-mGO (0.01 wt.%) in the linear regression (curve 2), it can be seen that the Person’s coefficient becomes 0.97, making the correlation between the tests very strong. Regardless of considering point A1 in the correlation, it can be concluded that there is a strong or very strong correlation between the ultimate tensile properties and the wear resistance of the nanocomposites produced here. It was previously seen that nanocomposites have high ultimate tensile properties due to the combined action of agglomerated and nonagglomerated mGO. Therefore, it can be concluded that these two components contained in the nanocomposites were also essential for the high wear resistance of the nanocomposites.

**Figure 5:**
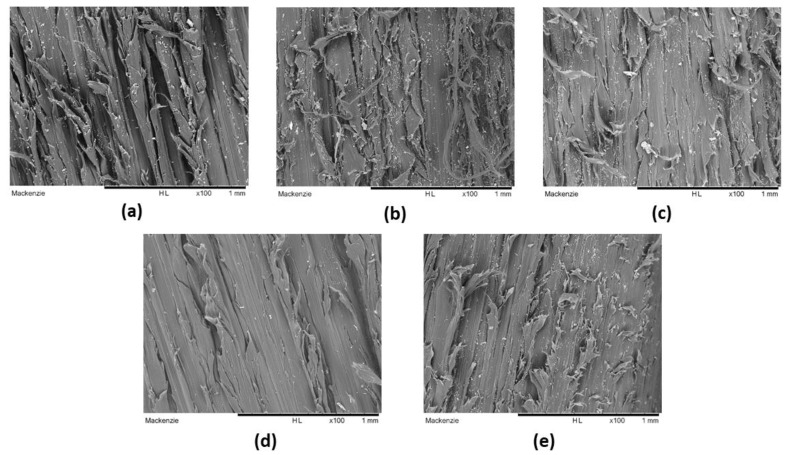
Images of the worn surface of the HMWPE (**a**), UHMWPE (**b**), HMWPE-UHMWPE (80/20 wt.%) (**c**), HMWPE-mGO (0.1 wt.%) (**d**) and HMWPE-UHMWPE (80/20 wt.%)-mGO (0.1 wt.%) (**e**) after the abrasive test.

Figure 5 shows the worn surface of the HMWPE, UHMWPE, HMWPE-UHMWPE (20 wt.%), HMWPE-mGO (0.1 wt.%) and HMWPE-UHMWPE (80/20 wt.%)-mGO (0.1 wt.%) after the abrasive test. As a result of the high wear of the HMWPE, Figure 5a shows that the worn surface of this sample is complete with deep grooves on the wear track. In the case of UHMWPE (Figure 5b), the deep grooves are not strongly present on the entire worn surface, due to their higher wear resistance. Unlike neat polymers, the worn surfaces of the HMWPE-UHMWPE (80/20 wt.%) (Figure 5c) blend and its nanocomposites (Figure 5d,e) present very shallow wear tracks, mainly the HMWPE-UHMWPE (80/20wt.%)-mGO (0.1 wt.%). The images of the wear surface corroborate the results of the abrasive tests previously observed.

**Figure 6:**
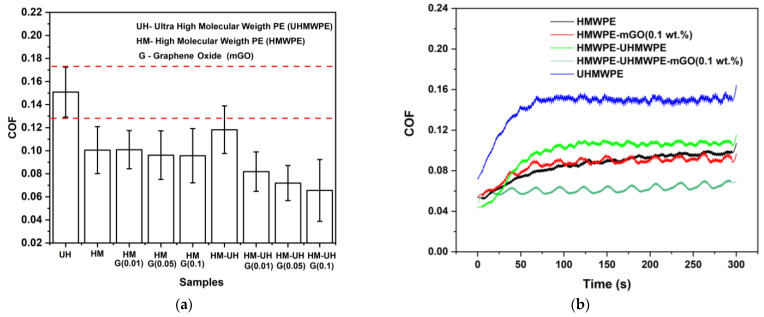
Friction coefficient results (COF) for the UHMWPE, HMWPE, HMWPE-mGO (0.01, 0.05 and 0.1 wt.%), HMWPE-UHMWPE (80/20 wt.%) and HMWPE-UHMWPE (80/20 wt.%)-mGO (0.01, 0.05 and 0.1 wt.%) (**a**) and the representative COF curves for the highlighted materials (**b**).

Figure 6a,b show the results of the friction coefficient of the studied materials (a) and the representative COF curves for some materials (b). They show that the HMWPE and UHMWPE have a solid lubricating behaviour due to their very low friction coefficient. This characteristic is extremely important for tribological applications [3]. Klapperich et al. (1999) reported that the self-lubricating characteristic of polyethylene is due to the ease of orientation of the amorphous and crystalline phases when shear stress is applied during the tribological test. They have attributed the orientation of the amorphous and crystalline phases to the amorphous phase deformation mechanism, the interlamellar slip (or shear) phenomenon [1]. Bowden and Young (1974) predicted that this mechanism involves the orientation of the lamella crystals parallel to each other, with the amorphous phase undergoing a simple shear deformation [41].

Figure 6 also indicates that UHMWPE has a higher friction coefficient than HMWPE. This can be explained by the different characteristics of the amorphous phase of each polymer. UHMWPE has an amorphous phase with higher molecular entanglement than HMWPE due to its higher molecular weight [23]. Due to this, the amorphous phase of the UHMWPE has a higher resistance to shear deformation (interlamellar slip phenomenon) and, consequently, a higher friction coefficient than the HMWPE. As a result, it can be observed that the addition of 20 wt.% of UHMWPE to the HMWPE (HMWPE-UHMWPE blend) tends to increase the friction coefficient of the HMWPE. On the other hand, analysing the nanocomposites, it is observed that the addition of mGO to the HMWPE and HMWPE-UHMWPE (80/20 wt.%) did not increase the coefficient of friction of the materials. The HMWPE-UHMWPE (80/20 wt.%)-mGO nanocomposites, on the contrary, showed a lower friction coefficient than the HMWPE, even containing the UHMWPE phases. This indicates that mGO contributes favourably to the interlayer slip phenomenon of the polymer. The friction coefficient reduction with the addition of mGO to the HMWPE-UHMWPE (80/20 wt.%) may be due to the superlubricity effect of the mGO agglomerates present in the nanocomposites. In other words, the flake–flake slipping of the agglomerates must be facilitating the deformation of the amorphous phase (interlayer slip phenomenon) of the polymer, contributing to the reduction in the polymer’s friction coefficient. The superlubricity that is suggested here is associated with the slipping of the agglomerates contained in the polymer, and not that the nanocomposites showed superlubricity, as note that the COF of the materials was around 0.01. It is important to note that all nanocomposites showed a lower friction coefficient than the UHMWPE.

Several authors have shown that the addition of 0.1 to 8 wt.% of carbon nanotubes, alumina nanoparticles, montmorillonite (clay) nanoparticles, graphene oxide, titanium dioxide and carbon nanofiber to UHMWPE allows one to obtain nanocomposites with wear resistances higher (up to 80%) than that of UHMWPE. However, many of the studied nanocomposites showed significant increases in the coefficient of friction (up to 120%) [7,36]. The nanocomposites developed in this work have two great advantages compared to the nanocomposites reported. The first is related to the fact that HMWPE nanocomposites presented a simultaneously higher wear resistance (up to 25%) and lower coefficient of friction (up to 56%) compared to UHMWPE. The second major advantage is related to the low filler content (<0.1 wt.%) used to develop the HMWPE nanocomposites, and values lower than 10 to 100% of filler content were used here. Therefore, this indicates that the use of HMWPE as a matrix for the development of high-performance materials is an excellent strategy.

Other authors have shown that hybrid composites containing nano- and micro-fillers have a better tribological performance than when the polymer contains a single filler [2,42]. Graphite and molybdenum dioxide microparticles are generally used to produce hybrid composites, given their lubricating properties [43,44]. Similar to the mGO agglomerates, both particles also display the superlubricity phenomenon [28,45]. However, the authors portrayed them only as self-lubricating particles and not based on the superlubricity phenomenon. This last concept was defended by Ferreira et al. (2019) [13] and Dienwiebel et al. (2004) [28] as the phenomenon that explains the self-lubricating properties of these particles. Therefore, it is reasonable to propose that the nanocomposites studied here resemble hybrid composites, with the mGO agglomerates acting like self-lubricating microparticles (graphite oxide [13]) and well-exfoliated mGO (nonagglomerated) as the nanoparticle. Therefore, as a suggestion for future work, the development of hybrid composites containing graphene oxide and graphite oxide or graphite can be a good strategy for the development of new materials. 

## 4. Conclusions

In view of the results presented, we can conclude that the HMWPE-mGO and HMWPE-UHMWPE (80/20 wt.%)-mGO nanocomposites developed here can be used as alternative materials to UHMWPE as the new materials showed a higher abrasive wear resistance and lower friction coefficient. Both features of the new materials are attractive for reducing maintenance costs, increasing the efficiency of machines, and improving the productivity of operations in the applications where the UHMWPE is used. The HMWPE-UHMWPE (80/20 wt.%)-mGO (0.1wt.%) nanocomposite was the material that presented the best tribological performance among the studied materials; this shows that adding the UHMWPE and mGO simultaneously to the HMWPE is a good strategy to achieve a high-performance material. It was observed that the presence of a large amount of mGO agglomerate in the polymer matrix, instead of being harmful, was crucial for the good tribological performance of the nanocomposites due to the superlubricity phenomenon.

## Figures and Tables

**Table 1:** Values of mechanical properties of the nanocomposites obtained from tensile test.

Samples	mGO (wt.%)	Elastic Modulus (MPa)	Stress at Breaking (MPa)	Strain at Breaking (%)	Reference
HMWPE	0	834.9 ± 94.8	37.7 ± 1.2	303.4 ± 13.3	[12]
	0.01	721.5 ± 134.3	44.3 ± 3.9	376.9 ± 34.4	-
	0.05	842.3 ± 43.6	41.2 ± 1.6	336.0 ± 17.7	-
	0.1	824.2 ± 70.0	43.4 ± 1.9	340.7 ± 11.7	[12]
HMWPE-UHMWPE (80/20 wt.%)	0	509.9 ± 52.5	42.0 ± 1.1	336.8 ± 15.7	[23]
	0.01	637.0 ± 97.0	40.6 ± 2.6	365.3 ± 28.1	-
	0.05	657.5 ± 68.2	40.6 ± 2.3	361.8 ± 42.6	-
	0.1	726.9 ± 82.2	41.5 ± 1.3	369.4 ± 1.9	-

## Data Availability

Data is contained within the article or Supplementary Materials.

## Supplementary Materials

The following are available online at https://www.mdpi.com/article/10.3390/polym13142237/s1, Figure S1: DSC curves of the HMWPE, HMWPE-UHMWPE (80/20 wt.%) blend and their nanocomposites, Table S1: Fusion enthalpy (ΔH) and the crystallinity degree (Xc) values of the HMWPE, HMWPE-UHMWPE, their nanocomposites and UHMWPE obtained from DSC analysis.

## References

[1] Klapperich C., Komvopoulos K., Pruitt L. (1999). Tribological properties and microstructure evolution of ultra-high molecular weight polyethylene. J. Tribol..

[2] Briscoe B.J., Sinha S.K., Friedrich K., Schlarb A.K. (2013). Tribological applications of polymers and their composites: Past, present and future prospects. Trybology of Polymeric Nanocomposites.

[3] Kurtz S.M. (2004). The UHMWPE Handbook: Ultra-High Molecular Weight Polyethylene in Total Joint Replacement.

[4] Kanagaraj S., Mathew M.T., Fonseca A., Oliveira M.S.A., Simões J.A.O., Rocha L.A. (2010). Tribological characterisation of carbon nanotubes/ultrahigh molecular weight polyethylene composites: The effect of sliding distance. Int. J. Surf. Sci. Eng..

[5] Tai Z., Chen Y., An Y., Yan X., Xue Q. (2012). Tribological behavior of UHMWPE reinforced with graphene oxide nanosheets. Tribol. Lett..

[6] Wei Z., Zhao Y.-P., Ruan S.-L., Gao P., Yu T.-X. (2006). A study of the tribological behavior of carbon-nanotube-reinforced ultrahigh molecular weight polyethylene composites. Surf. Interface Anal..

[7] Puértolas J.A., Kurtz S.M. (2014). Evaluation of carbon nanotubes and graphene as reinforcements for UHMWPE-based composites in arthroplastic applications: A review. J. Mech. Behav. Biomed. Mater..

[8] Pelto J., Verho T., Ronkainen H., Kaunisto K., Metsäjoki J., Seitsonen J., Karttunen M. (2019). Matrix morphology and the particle dispersion in HDPE nanocomposites with enhanced wear resistance. Polym. Test..

[9] Hwang S.-S., Hsu P.P., Yeh J.-M., Yang J.-P., Chang K.-C., Lai Y.-Z. (2009). Effect of clay and compatibilizer on the mechanical/thermal properties of microcellular injection molded low density polyethylene nanocomposites. Int. Commun. Heat Mass Transf..

[10] Li K., Tjong S.C. (2011). Preparation and mechanical and tribological properties of high-density polyethylene/hydroxyapatite nanocomposites. J. Macromol. Sci. Part B Phys..

[11] Ferreira E.H.C., Fechine G.J.M. (2021). High abrasive wear resistance polyethylene blends: An adapted Ratner-Lancaster correlation. Polym. Bull..

[12] Ferreira E.H.C., de Lima L.P., Fechine G.J.M. (2020). The “Superlubricity State” of Carbonaceous Fillers on Polymer Composites. Macromol. Chem. Phys..

[13] Ferreira E.H.C., Andrade R.J.E., Fechine G.J.M. (2019). The “superlubricity State” of Carbonaceous Fillers on Polyethylene-Based Composites in a Molten State. Macromolecules.

[14] Yip F., Diraddo R., Hatzikiriakos S.G. (2000). Effect of combining boron nitride with fluoroelastomer on the melt fracture of HDPE in extrusion blow molding. J. Vinyl Addit. Technol..

[15] Kazatchkov I.B., Yip F., Hatzikiriakos S.G. (2000). The effect of boron nitride on the rheology and processing of polyolefins. Rheol. Acta.

[16] Muñoz P.A.R., de Oliveira C.F.P., Amurin L.G., Rodriguez C.L.C., Nagaoka D.A., Tavares M.I.B., Domingues S.H., Andrade R.J.E., Fechine G.J.M. (2018). Novel improvement in processing of polymer nanocomposite based on 2D materials as fillers. Express Polym. Lett..

[17] Durighetto Y., Amurin L.G., Valim F.F., Fechine G.J.M., Andrade R.J.E. (2019). The role of physical structure and morphology on the photodegradation behaviour of polypropylene-graphene oxide nanocomposites. Polymer.

[18] Vega J.F., Martínez-Salazar J., Trujillo M., Arnal M.L., Müller A.J., Bredeau S., Dubois P. (2009). Rheology, processing, tensile properties, and crystallization of polyethylene/carbon nanotube nanocomposites. Macromolecules.

[19] Khasraghi S.S., Rezaei M. (2013). Preparation and characterization of UHMWPE/HDPE/MWCNT melt-blended nanocomposites. J. Thermoplast. Compos. Mater..

[20] Bhusari S.A., Sharma V., Bose S., Basu B. (2019). HDPE/UHMWPE hybrid nanocomposites with surface functionalized graphene oxide towards improved strength and cytocompatibility. R. Soc. Interface.

[21] Song J., Yang W., Fu F., Zhang Y. (2014). The Effect of Graphite on the Water Uptake, Mechanical Properties, Morphology, and EMI Shielding Effectiveness of HDPE/Bamboo flour composites. BioResources.

[22] Cao C., Daly M., Chen B., Howe J.Y., Singh C.V., Filleter T., Sun Y. (2015). Strengthening in Graphene Oxide Nanosheets: Bridging the Gap between Interplanar and Intraplanar Fracture. Nano Lett..

[23] Ferreira E.H.C., Fechine G.J.M. (2020). Healing phenomenon adapted to understand the miscibility of polymer blends: An approach based on the deformation mechanism. J. Appl. Polym. Sci..

[24] Lucas A.D.A., Ambrósio J.D., Otaguro H., Costa L.C., Agnelli J.A.M. (2011). Abrasive wear of HDPE/UHMWPE blends. Wear.

[25] Sheldon R.P. (1963). Density and Degree of Crystallinity in Polymers. J. Polym. Sci. Part B Polym. Lett..

[26] Murphy J. (2001). Additives for Plastics Handbook.

[27] Bartczak Z., Galeski A. (2014). Mechanical properties of polymer blends. Polymer Blends Handbook.

[28] Dienwiebel M., Verhoeven G.S., Pradeep N., Frenken J.W.M., Heimberg J.A., Zandbergen H.W. (2004). Superlubricity of Graphite. Phys. Rev. Lett..

[29] Dienwiebel M., Pradeep N., Verhoeven G.S., Zandbergen H.W., Frenken J.W.M. (2005). Model experiments of superlubricity of graphite. Surf. Sci..

[30] Verhoeven G.S., Dienwiebel M., Frenken J.W.M. (2004). Model calculations of superlubricity of graphite. Phys. Rev. B.

[31] Kalaitzidou K., Fukushima H., Drzal L.T. (2007). Mechanical properties and morphological characterization of exfoliated graphite-polypropylene nanocomposites. Compos. Part A Appl. Sci. Manuf..

[32] Crist B., Fisher C.J., Howard P.R. (1989). Mechanical Properties of Model Polyethylenes: Tensile Elastic Modulus and Yield Stress. Macromolecules.

[33] Young R.J., Kinloch I.A., Gong L., Novoselov K.S. (2012). The mechanics of graphene nanocomposites: A review. Compos. Sci. Technol..

[34] Penu C., Hu G.-H., Fernandez A., Marchal P. (2012). Lionel Choplin Rheological and Electrical Percolation Thresholds of Carbon Nanotube/Polymer Nanocomposites. Polym. Eng. Sci..

[35] Khruschov M.M. (1974). Principles of abrasive wear. Wear.

[36] Xu S., Tangpong X.W. (2013). Review: Tribological behavior of polyethylene-based nanocomposites. J. Mater. Sci..

[37] Lancaster J.K. (1968). Relationships Between the Wear of Polymers and their Mechanical Properties. Proc. Inst. Mech. Eng. Conf. Proc..

[38] Kanagaraj S., Varanda F.R., Zhil’tsova T.V., Oliveira M.S.A., Simões J.A.O. (2007). Mechanical properties of high density polyethylene/carbon nanotube composites. Compos. Sci. Technol..

[39] Srinath G., Gnanamoorthy R. (2006). Two-body abrasive wear characteristics of Nylon clay nanocomposites-effect of grit size, load, and sliding velocity. Mater. Sci. Eng. A.

[40] Morioka Y., Tsuchiya Y., Shioya M. (2015). Correlations between the abrasive wear, fatigue, and tensile properties of filler-dispersed polyamide 6. Wear.

[41] Bowden P.B., Young R.J. (1974). Deformation mechanisms in crystalline polymers. J. Mater. Sci..

[42] Bahadur S., Schwartz C.J., Briscoe C.J. (2008). The influence of nanoparticle fillers in polymer matrices on the formation and stability of transfer film during wear. Tribology of Polymeric Nanocomposites.

[43] Cenna A.A., Dastoor P., Beehag A., Page N.W. (2001). Effects of graphite particle addition upon the abrasive wear of polymer surfaces. J. Mater. Sci..

[44] Li C., Zhang Z., Ye L., Friedrich K. (2008). Synergistic effects of nanoparticles and traditional tribo-fillers on sliding wear of polymeric hybrid composites. Tribology of Polymeric Nanocomposites: Friction and Wear of Bulk Materials and Coatings.

[45] Martin J.M., Donnet C., Mogne T.L., Epicier T. (1993). Superlubricity of molybdenum disulphide. Phys. Rev. B.

