# Supplementary material for: High-Tribological-Performance Polymer Nanocomposites: An Approach Based on the Superlubricity State of the Graphene Oxide Agglomerates
Source: Polymers (Basel). 2021 Jul 8;13(14):2237. doi: 10.3390/polym13142237 (PMC8309333; doi:10.3390/polym13142237)
Supplement: Supplementary file 1 [file polymers-13-02237-s001.zip › polymers-1277340-supplementary.pdf]

# Supporting Information

# High tribological performance polymer nanocomposites: An approach based on the superlubricity state of the oxide graphene agglomerates

*Eder. H. C. Ferreira[1], Angela Aparecida Vieira[2], Lúcia Vieira[2], Guilhermino J. M. Fechine*[1]*

*[1] Mackenzie Institute for Research in Graphene and Nanotechnologies - MackGraphe. Mackenzie Presbyterian University. Rua da Consolação. 896. São Paulo - SP. 01302-907. Brazil*

*2 Institute of Research and Development - IP&D University of Paraiba Valley -Univap, Av. Shishima Hifumi, 2911 - Urbanova, São José dos Campos - SP 12244-000 Brazil*

**RESULTS AND DISCURSIONS**

Figure S1 shows the DSC curves of the HMWPE, HMWPE-mGO (0.01, 0.05 and 0.1 wt.%), HMWPE-UHMWPE (80/20 wt.%), HMWPE-UHMWPE (80/20 wt.%)-mGO (0.01, 0.05 and 0.1 wt.%) and UHMWPE.

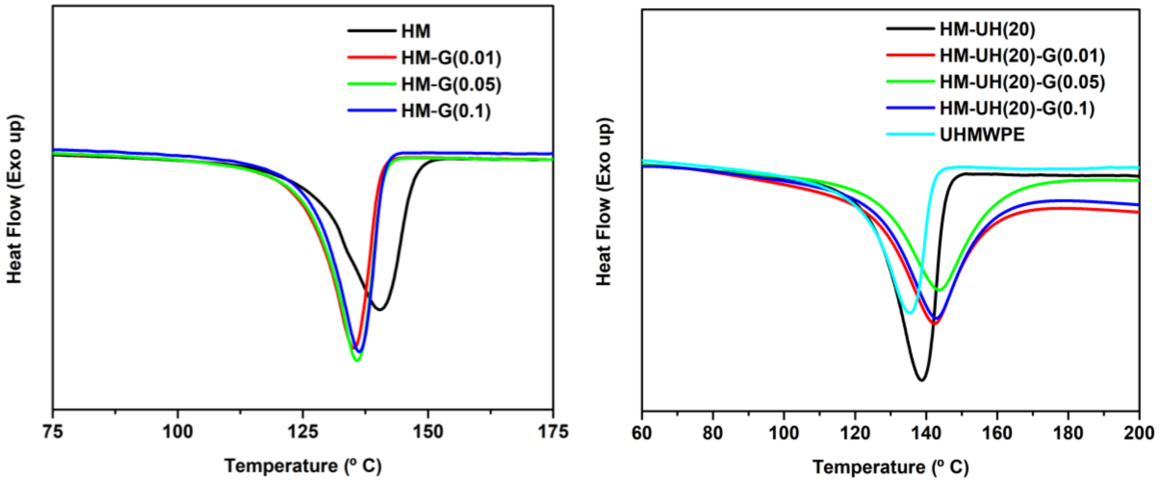


a) b)

Figure S1. DSC curves of the HMWPE, HMWPE-UHMWPE (80/20 wt.%) blend and their nanocomposites.

Table S1 shows the fusion enthalpy (ΔH) and the crystallinity degree (Xc) of the HMWPE, HMWPE-mGO (0.01, 0.05 and 0.1 wt.%), HMWPE-UHMWPE (80/20 wt.%), HMWPE-UHMWPE (80/20 wt.%)-mGO (0.01, 0.05 and 0.1 wt.%) and UHMWPE obtained from DSC analysis.

**Table S1.** Fusion enthalpy (ΔH) and the crystallinity degree (Xc) values of the HMWPE, HMWPE-UHMWPE, their nanocomposites and UHMWPE obtained from DSC analysis

| **Samples** | **ΔH (J/g)** | **Xc (%)** |
|---|---|---|
| HMWPE | 169.79 | 66.5 |
| HMWPE-mGO (0.01 wt.%) | 154.11 | 60.5 |
| HMWPE-mGO (0.05 wt.%) | 163.61 | 64.16 |
| HMWPE-mGO (0.1 wt.%) | 160.97 | 63.20 |
| HMWPE-UHMWPE (20 wt.%) | 151.95 | 59.58 |
| HMWPE-UHMWPE (80/20 wt.%)-mGO (0.01 wt.%) | 154.73 | 60.67 |
| HMWPE-UHMWPE (80/20 wt.%)-mGO (0.05 wt.%) | 159.67 | 62.61 |
| HMWPE-UHMWPE (80/20 wt.%)-mGO (0.1 wt.%) | 161.10 | 63.17 |
| UHMWPE | 111.71 | 44.25 |

The crystallinity degree (Xc) showed in Table 1S was calculate using the Equation 2, where $\Delta H^*$ is the fusion enthalpy of 100 % crystalline polymer (293 J/cm$^3$)[25,35] and $\Delta H$ is the fusion enthalpy measured using DSC data.

$$Xc = \frac{\Delta H}{\Delta H^*} x100 \qquad 1S$$
